# New Stx2e Monoclonal Antibodies for Immunological Detection and Distinction of Stx2 Subtypes

**DOI:** 10.1371/journal.pone.0132419

**Published:** 2015-07-20

**Authors:** Craig Skinner, Stephanie Patfield, Bradley J. Hernlem, Xiaohua He

**Affiliations:** Western Regional Research Center, U.S. Department of Agriculture, Agricultural Research Service, 800 Buchanan Street, Albany, California, United States of America; Beijing Institute of Microbiology and Epidemiology, CHINA

## Abstract

**Background:**

Stx2e is a primary virulence factor in STEC strains that cause edema disease in neonatal piglets. Though Stx2a and Stx2e are similar, many antibody-based Stx detection kits are designed to detect Stx2a and do not recognize the Stx2e subtype.

**Methods and Findings:**

Four monoclonal antibodies against Stx2e were developed and characterized. Two of these mAbs recognize the B subunit of Stx2e, Stx2f, and to a lesser extent, Stx2b, Stx2c, and Stx2d. The other two mAbs recognize the A subunit of Stx2e, and cross-react with all Stx2 subtypes except Stx2f. The most sensitive sandwich ELISA using these mAbs has a limit of detection for Stx2e of 11.8 pg/mL. The ability of the neutralizing antibody Stx2e-2 to block Stx2e-receptor binding in Vero cells was visualized using immunofluorescence. Combinations of these and previously developed mAbs permit ELISA-based differentiation between closely related Stx2a, Stx2c, and Stx2d (using mAbs Stx2-5/2-1, Stx2-5/2e-2, and Stx2e-3/2e-2, respectively).

**Conclusions:**

The sensitive immunoassays developed in this study should augment our capacity to detect Stx2e in porcine environments and biological samples. Moreover, immunoassays that can distinguish between the closely related Stx2a, Stx2c, and Stx2d subtypes can be useful in quickly analyzing Stx subtypes in samples containing more than one strain of STEC.

## Introduction

Strains and serotypes of Shiga toxin-producing *Escherichia coli* (STEC) that express Shiga toxin 2e (Stx2e) are a major cause of edema disease in newly weaned piglets worldwide [1], which can result in internal bleeding, paralysis, and death for infected animals. There is currently no widely used treatment for edema disease, and once symptoms appear the disease has often progressed to an advanced state. Stx2e-expressing STEC strains have been found in human patients as well, but symptoms associated with these infections are usually mild [2–4].

Stx2e, like all other Stxs and several other bacterial AB_5_ toxins, consists of a single catalytic A subunit and a homo-pentameric B subunit [5]. The B subunit pentamer is required for receptor binding on the surface of target cells, while the A subunit, an *N*-glycosidase, inactivates ribosomes by removing a specific nucleotide from the 28S rRNA [6]. The cellular receptor for Stx2e is thought to be Gb4, although it can also bind to Gb3 [7, 8]. This receptor preference is shared by only one other Stx2 subtype, Stx2f, and may contribute to potentially lethal complications in neonatal pigs, but relatively mild disease in humans. The receptor for all other Stx subtypes is Gb3, though Stx2a has been shown to recognize Gb4 in some studies but not in others [8, 9].

Immunoassays are a highly effective method of detecting Stx2 subtypes [10, 11], and high-affinity antibodies could be used for detection of Stx2e as well, or even as a prophylaxis or treatment for edema disease. Stx2e has a high homology to Stx2a, in both its mature A (93.9% at the amino acid level) and mature B subunits (86.8% at the amino acid level). Some antibodies that recognize the A subunit of Stx2a cross-react with Stx2e [12]; however, few commercial ELISA kits recognize Stx2e at low levels [11] and no monoclonal antibodies (mAbs) that recognize the B subunit of Stx2e have been characterized. B subunit-specific antibodies are particularly useful because they often have the ability to neutralize the toxicity of Stxs [13–15]. Additionally, there are no commercially available Stx2e-specific antibodies, although single chain antibodies have been reported [16]. Therefore, there is a need for sensitive, specific monoclonal antibodies that recognize Stx2e and are compatible with immunoassays, especially against the Stx2e B subunit.

Many STEC strains express more than one type or subtype of Stx, with Stx1/Stx2a, Stx2a/Stx2c, and Stx2a/Stx2d being the most common, and strains expressing multiple Stx are frequently found in disease-causing serotypes such as O157:H7 [17]. Mass spectrometry is primarily used to distinguish Stx2a from Stx2c and Stx2d in a bacterial culture or stool sample [18, 19]: since Stx2a, Stx2c, and Stx2d are very similar at the amino acid level, few mAbs have been verified to recognize one of the three specifically, although mAbs that can neutralize Stx2a but not Stx2c have been reported [20]. However, mass spectrometry is inconvenient as a bench top assay method and only measures toxin fragments rather than intact toxin. Here, we report four new anti-Stx2e mAbs, which in combination with each other or previously developed anti-Stx2 mAbs, are capable of not only detecting Stx2e at low concentrations, but also distinguishing the very closely related Stx2 subtypes Stx2a, Stx2c, and Stx2d.

## Materials and Methods

### Ethics statement

All procedures with animals were carried out according to institutional guidelines for husbandry approved by the Institutional Animal Care and Use Committee of the U.S. Department of Agriculture, Western Regional Research Center (USDA IACUC). This specific procedure and protocol was reviewed and approved by the USDA IACUC (Protocol# 09-J-10). Mice were euthanized using rapid cervical dislocation to minimize suffering.

### E. coli strains and growth conditions

Strains expressing Stx1a (RM13506), Stx2a (RM10638), Stx2b (RM7005), Stx2c (RM10058), Stx2d (RM8013), Stx2e (RM7958), Stx2f (RM7007), Stx2g (RM10468), and a pathogenic *E*. *coli* O6 strain that doesn’t express Stx (ATCC 25922) were grown as described [8]. Autoclaved LB medium was inoculated with a bacterial strain, which was grown overnight (12 hours) at 37°C at 150 rpm, and then inoculated at a 50-fold dilution into fresh LB medium supplemented with 50 ng/mL mitomycin C, and this culture was grown 16 hours in a 37°C shaking incubator. The cultures were centrifuged, and the supernatant was filter-sterilized (0.2 μm). A list of all strains used is included in Table 1.

**Table 1:** *E*. *coli* strains used in this study.

Strain	Other names	Serotype	Stx2 subtype expressed	Origin	Reference
RM10638		O157:H7	Stx2a	Cow (2009)	[23]
RM7005	EH250	O188:H12	Stx2b	Clinical	[23]
RM10058		O157:H7	Stx2c	Bird (2009)	[23]
RM8013		ND^a^	Stx2d	Cow (2008)	[23]
RM7988		ND^a^	Stx2e	Water (2008)	[8]
RM7007	T4/97	O128:H2	Stx2f	Feral pigeon	[23]
RM10468		ND^a^	Stx2g	Cow (2009)	[23]
RM13506		O45	Stx1a	Human	[17]
25922 (ATCC)	Seattle, 1946	O6	No toxin	Clinical	[21]
CWG308 pJCP-Gb3			Gb3-LPS		[24]
CWG308 pJCP-*lgt*CDE			Gb4-LPS		[25]
CWG308					[24]
BL21 (DE3) Stx2e (E167Q)			Stx2e (E167Q)		This study
TOP10					Invitrogen
TOP10 pTrcHis2-Stx2e-B			Stx2e B subunit +6xHis		This study

^a^ Not determined.

### Cloning, expression, and purification of Stx2e (E167Q) toxoid

To produce a non-catalytic toxoid for mouse immunization, the E167Q point mutation was introduced into the A subunit of Stx2e using mutagenic PCR [10]. The *stx2e* (E167Q) operon (including both the A and B subunits of Stx2e) was constructed by mutagenic PCR and incorporated into the pQE-T7-2 vector by methods previously described [10]. This construct was then transformed into BL21 (DE3) pLysS competent cells, grown for 12 hours in LB with 50 μg/mL kanamycin at 30°C and 150 rpm, and diluted 50-fold into 500 mL LB plus 50 μg/mL kanamycin. This was grown for 4 hours at 30°C, then induced with 1 mM IPTG (isopropyl β-D-1-thiogalactopyranoside, Sigma-Aldrich) overnight at 20°C. Cells were centrifuged (at 5000 G for 15 min., 4°C), resuspended in PBS, and sonicated. Cell debris was centrifuged and discarded (5000 G, 15 min. at 4°C), while MnCl_2_ was added to the lysate at a final concentration of 50 mM. This mixture was stirred at room temperature (RT, 22°C) for 10 min., then centrifuged (5000 G, 30 min. 4°C), and the debris was discarded. NH_4_SO_4_ was added to the lysate at 60% saturation, and protein was allowed to precipitate for 15 minutes while stirring on ice. Precipitated protein was then centrifuged (5000 G, 30 min., 4°C) and resuspended in PBS. This was buffer exchanged to 50 mM NaPO_4_, pH 6.7 using a Zeba desalting column (Fisher Scientific). Cation exchange was performed using an Akta FPLC and a HiTrap SP-HP column (GE Healthcare) and Stx2e (E167Q) was eluted using 50 mM NaPO_4_, pH 7.6 plus 1 M NaCl. Positive fractions were concentrated by Amicon centricon (Fisher Scientific), and subjected to gel filtration on a Sephadex 100 HiPrep column with PBS buffer.

### Immunization, splenocyte extraction, and cell culture

SP2/0 myeloma cells and splenocytes were grown and prepared as previously described [8]. Mouse immunizations were performed using Stx2e (E167Q) toxoid in the Sigma adjuvant system (Sigma-Aldrich). Mice received 5 μg of Stx2e (E167Q)/adjuvant mix per intraperitoneal injection at two-week intervals for a total of three injections. Two weeks after the third injection, mice were boosted with 1 μg/mouse Stx2e (E167Q) in sterile PBS. Four days later, mice were sacrificed by rapid cervical dislocation, spleens were excised aseptically, and splenocytes were harvested as previously described [8].

### Fusion, cloning, and screening of hybridomas

Monoclonal antibodies (mAbs) were produced as described [8]. Briefly, cell fusions were achieved using SP2/0 myeloma cells, splenocytes extracted from the inoculated mouse spleen, and a polyethylene glycol-based protocol. Clonal hybridoma lines were then achieved with three rounds of cloning by limited dilution, regrowth, and screening.

### Monoclonal antibody preparation

Monoclonal antibodies were produced and purified by previously described methods [8, 21]. Briefly, clonal hybridoma lines were grown in complete hybridoma medium (Iscove’s modified Dulbecco’s Minimal medium [Sigma Aldrich] containing NaHCO_3_ [36 mM] and 1x Glutamax [Invitrogen, Calsbad, CA], supplemented with 10% heat-inactivated fetal calf serum [FCS] [Invitrogen]). The purification of monoclonal antibody was conducted as described [8]. Briefly, 250–450 mL of antibody-containing media (hybridoma cells grown for 4–5 days) was passed through a Protein G column (GE Healthcare). Antibody was eluted with 0.1 M glycine (pH 2.7), resulting in 3–11 mg of purified Stx2e antibody. Protein concentration was determined using the BCA Protein Assay Kit (Thermo Scientific, Rockford, IL). Biotinylation of antibodies was conducted using the Lightning-Link Biotin Conjugation Kit (Innova Biosciences, Cambridge, UK). Antibodies were isotyped by ELISA using Stx2e (E167Q) toxoid and horseradish peroxidase (HRP)-conjugated isotype-specific antibodies (Southern Biotech, Birmingham, AL).

### Enzyme-linked immunosorbent assays (ELISAs)

Hybridoma screening ELISAs were conducted as previously described [8]. Briefly, 50 ng/mL Stx2e (E167Q) toxoid in PBS was incubated overnight at 4°C in the wells of black Nunc Maxisorp ELISA plates. The plates were washed with PBS/0.05% Tween 20 (PBST), blocked with 5% nonfat dry milk/PBST, washed again, then a combination of 50 μL/well blocking solution and 50 μL/well hybridoma medium was added, mixed, and incubated for 1 hour at RT. Following this, the plates were washed, and HRP-conjugated goat anti-mouse IgG antibody (GAM-HRP [Promega]) in blocking solution at a 1/5000 dilution was dispensed into the plates, and incubated for 1 hour at RT. The plates were again washed, and developed using Pico chemiluminescent substate (Thermo Scientific). Luminescence was measured using a Victor 3 plate reader (Perkin Elmer). A BioTek ELx405 plate washer was used for all washing steps. ELISAs were conducted thrice, with the exception of the hybridoma screening ELISAs (once), and a representative ELISA is shown. All data points on ELISAs in the figures were performed in triplicate or quadruplicate for proper standard deviation calculations.

For sandwich ELISAs, coating antibody was diluted in PBS to 1 μg/mL, and then 100 μL/well was allowed to bind to black Maxisorb plates overnight at 4°C. This was followed by washing twice with PBST, and then 200 μL/well blocking solution (3% BSA in PBST) was added and incubated for 1 hour at RT. The indicated purified toxin/toxoid or a 10-fold dilution of cell-free medium, diluted in PBS, was then added incubated at RT for 1 hour. The plates were washed six times with PBST, then the indicated biotinylated secondary antibody (0.5 μg/mL, diluted in BSA/PBST) was added and incubated for 1 hour at RT. The plates were washed a further six times, 0.2 μg/mL streptavidin-HRP (SA-HRP [Invitrogen]), diluted in BSA/PBST, was added, and the plates were incubated for 1 hour at RT. Following this, the plates were washed a final six times and developed using Pico chemiluminescent substrate (Thermo Scientific). Luminescence was measured using a Victor 3 plate reader. All sandwich ELISAs were conducted thrice for confirmation. Limit of detection (LOD) was calculated by extrapolating ng/mL of Stx2e (E167Q) from the background luminescence plus 3 standard deviations of the background.

### Western immunoblots

Western immunoblots were conducted as previously described [7]. Pure toxin/toxoid and cell-free medium samples were incubated at 72°C for 10 minutes in 1x NuPage SDS loading buffer, then run on a 4%–12% NuPAGE Novex Bis-Tris mini gel (Invitrogen). The proteins were then transferred to a PVDF membrane (pore size, 0.45 μm; Amersham Hybond-P), blocked with 2% ECL Prime blocking agent (GE Healthcare) in PBST, and washed with PBST (3x). Monoclonal antibodies were diluted to 1 μg/mL in blocking solution and incubated with the blots for 1 hour at RT, then the blots were washed again (3x) in PBST. GAM-HRP antibody (Promega) at a 1/20,000 dilution was incubated on the blot for 1 hour at RT. The blots were washed four more times with PBST (5 minutes each), and developed using Lumigen TMA-6 (Lumigen) substrate. The blots were visualized with a 5 minute exposure using a FluorChem HD2 (Alpha Innotech). All westerns were conducted three times.

### Antibody affinity measurements

Antibody affinity to Stx2e (E167Q) was measured using an Octet QK system (Forte-bio, Menlo Park, CA) as previously described [8]. Briefly, biotinylated mAbs were bound to streptavidin biosensors at 10 μg/mL, diluted in PBS. Stx2e (E167Q) was then incubated with the sensors at four different concentrations (142, 71, 36, and 18 nM) and then allowed to dissociate in PBS. Dissociation constants (K_D_) were calculated using the Octet QK software (Data Acquisition 7.0).

### Vero cell cytotoxicity assays

Vero cells were prepared and grown as described [8]. Briefly, the medium used for Vero cell propagation and growth was Dulbecco’s Modified Eagle Medium (DMEM, Invitrogen) with 10% fetal bovine serum (FBS) (Invitrogen), within a humidified cell culture incubator (37°C, 5% CO2). Before conducting the cytotoxicity assays, the cells were trypsinized, diluted to 10^5^ cells/mL, then dispensed into 96-well cell-culture-treated plates, and these plates were incubated for 24 hours. Filter-sterilized mitomycin-induced STEC culture medium was diluted in fresh Vero media (10 μL media/well for cell-free medium). mAbs were pre-incubated at 10 μg/mL with toxin for 1 hour at RT before adding the mixture to the Vero cells. The medium in the Vero cell assay plate was then removed and replaced with the Stx and/or mAb-containing mixture (100 μL/well). Twenty-four hours after treatment, the Vero cells were lysed using 100 μl/well of CellTitre-Glo reagent (Promega) diluted 1:2 in PBS, with 3 minutes of shaking. Luminescence was measured using a Victor II plate reader. All Vero cell toxicity assays were conducted three times with similar results. Photographs were obtained from replicate wells using a Leica microscope at 400x magnification.

### FACS analysis

Control cells and cells that express Gb3-LPS or Gb4-LPS were first prepared for FACS analysis. 20 μL of cells at 1 OD resuspended in PBS were centrifuged (12,000 rpm, for 2 minutes) and blocked for 1 hour in 100 μL of 3% BSA/PBS-Tween 20 (0.05%) at RT. Following this, cells were centrifuged and washed in PBS (1 mL) and incubated in 100 μL of 2 μg/mL Stx2e (E167Q) for 1 hour at RT. Cells were centrifuged and washed with PBS twice, and then incubated in 100 μL of 1 μg/mL mAb Stx2e-3 for 1 hour at RT. Cells were centrifuged and washed with PBS twice, then incubated with 100 μL of 1 μg/mL DyLight 488-conjugated goat anti-mouse antibody (Fisher Scientific). Cells were washed twice more, then analyzed with a FACS Vantage SE (BD Bioscience), using previously published parameters and employing a customized 491 nm Calypso laser (Cobolt) [22]. Data for 50,000 cells was collected for each sample.

### Stx2e immunofluorescence

Vero cells were seeded in 24-well plates at 10^5^ cells/mL. Cells were grown for 12 hours, then washed twice with PBS. For the samples in which cells were treated with Stx2e (E167Q) before fixing, cells were first treated with Stx2e (E167Q) at 1 μg/mL for 1 hour at 37°C in a cell culture incubator, washed with PBS, and then fixed to the culture plate with 4% paraformaldehyde for 30 minutes at RT. For samples in which cells were fixed before Stx2e (E167Q) treatment, cells were fixed first, then treated with Stx2e (E167Q) at the same concentrations, times, and temperatures. After Stx2e (E167Q) treatment and fixing, cells were then washed again with PBS and blocked with 3% BSA in PBST for 1 hr. at RT. Cells were then incubated with mAb Stx2e-3 at 1 μg/mL for 1 hr at RT. Following another two PBS washes, cells were incubated with 1 μg/mL goat anti-mouse DyLight for 1 hr. at RT. Cells were then washed twice with PBS and a 1 μg/mL solution of DAPI was added for 5 minutes. Cells were then observed and photographed using a Zeiss microscope under 400x magnification, and fluorescence was observed by exciting the DyLight conjugate with a broad-spectrum light. As the camera attached to this microscope only captures images in monochrome, the black and white DAPI and DyLight exposure photographs were artificially changed to blue and green using the GIMP2 program. Overlay images were also developed using GIMP2.

## Results

### Generation of Stx2e-binding monoclonal antibodies and their subunit specificities

**Fig 1:**
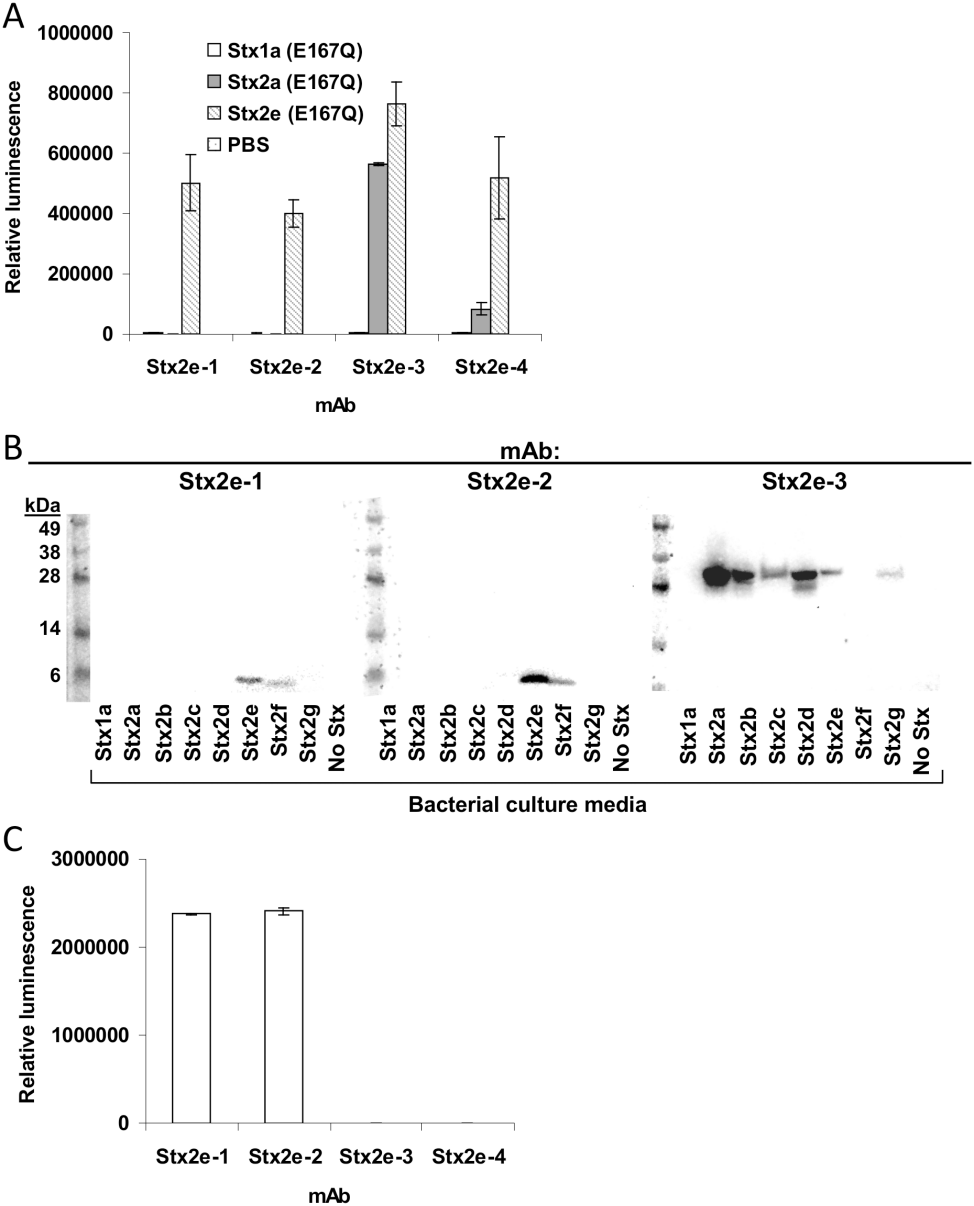
Stx2e antibody specificity. A) Direct ELISA using Stx2e mAbs and purified Stx toxoids. Stx2e mAb was added at 1 μg/mL; purified toxoids at 100 ng/mL. B) Western immunoblots for mAbs Stx2e-1, Stx2e-2, and Stx2e-3. Media from Stx-expressing strains were loaded at 10 μL/lane. The “No Stx” sample contains media from an *E*. *coli* strain that doesn’t express Stx (ATCC 25922). C) Stx2e B subunit ELISA. Direct ELISA with Stx2e B subunit bound to ELISA plates at 1 μg/mL. Antibodies were also used at 1 μg/mL.

Recombinant catalytically-inactive Stx2e toxoid harboring the E167Q point mutation was generated, expressed in *E*. *coli*, and purified by column chromatography (a listing of all bacterial strains used and generated in this study is provided in Table 1). This antigen was injected into mice and, using standard hybridoma techniques, splenocytes were extracted and fused to SP2/0 myeloma cells. A total of 1,920 wells of hybridomas were screened for antibodies that recognize Stx2e. After three rounds of clonal selection and recovery, four hybridomas that produced high-affinity monoclonal antibodies (mAbs) to Stx2e were isolated (Fig 1A). The isotype and dissociation constants for these mAbs are displayed in Table 2. mAbs Stx2e-1 and Stx2e-2 recognize the B subunits of Stx2e and Stx2f in a western immunoblot (Fig 1B), although they both can also detect Stx2b, Stx2c, and Stx2d in same-antibody sandwich ELISAs (Stx2e-1 or Stx2e-2 used for both capture and detection) (S1 Fig). mAb Stx2e-3 detects the A-subunit of all Stx2 subtypes except for Stx2f (Fig 1B). mAb Stx2e-4 did not recognize any Stx subtypes, even Stx2e, when analyzed by western immunoblot (data not shown). However, it does recognize Stx2e (E167Q) in a direct ELISA, as do the other three Stx2e mAbs (Fig 1A). Most likely, mAb Stx2e-4 recognizes a conformational epitope on Stx2e which is disrupted during SDS treatment. mAb Stx2e-4 does not recognize recombinant Stx2e B subunit (Fig 1C) in an ELISA, meaning that this is most likely an A subunit-specific antibody.

**Table 2:** Properties of Stx2e mAbs.

Antibody	Isotype	K_D_ (x 10^−9^ M)
Stx2e-1	IgG1, kappa	0.197 ± 0.048
Stx2e-2	IgG1, kappa	0.212 ± 0.019
Stx2e-3	IgG1, kappa	0.043 ± 0.020
Stx2e-4	IgG2b, kappa	1.220 ± 0.007

### Establishment of ELISAs for sensitive detection and distinction of Stx2 subtypes

**Fig 2:**
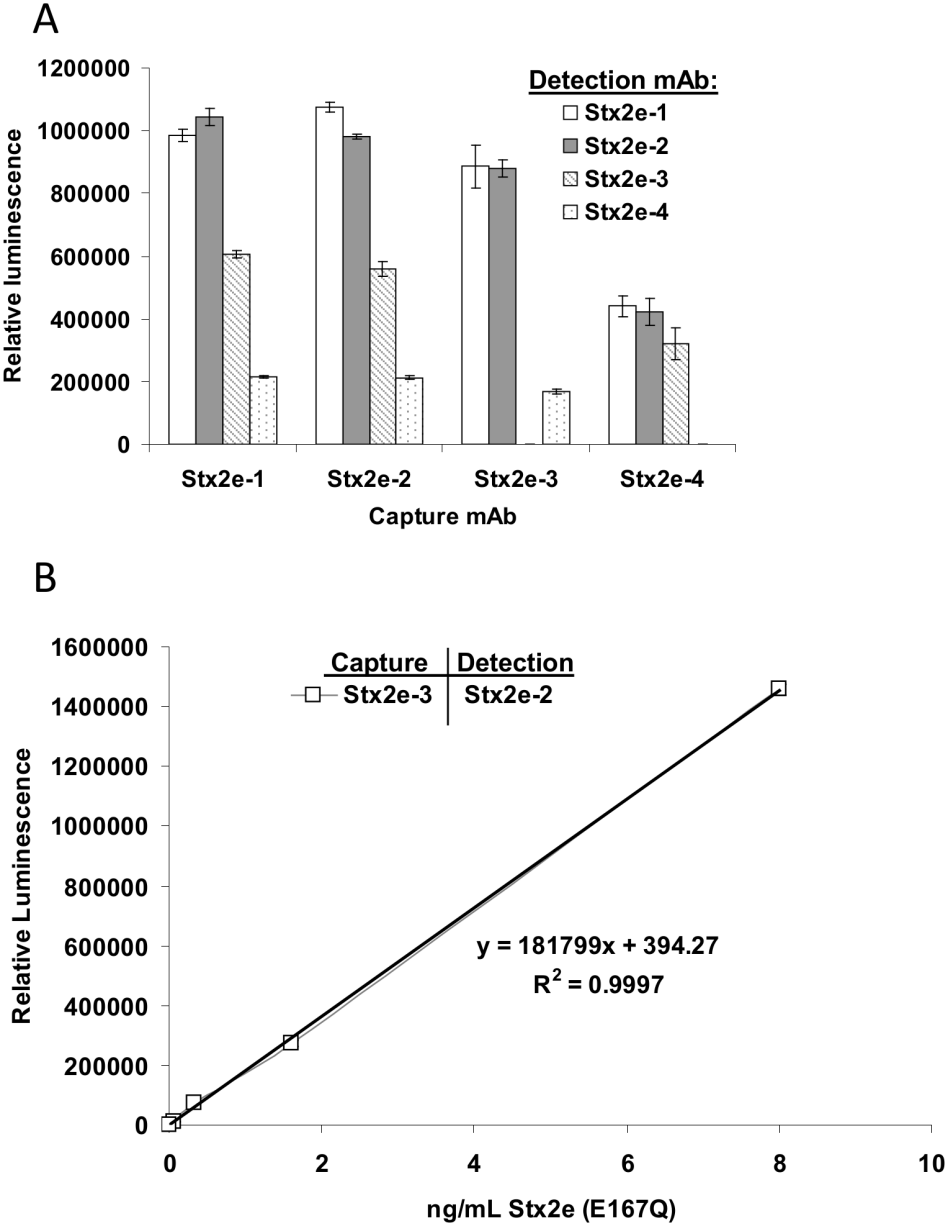
Stx2e ELISA sensitivity and specificity. A) Optimizing sandwich ELISA antibody combinations. Antibodies were used at 1 μg/mL for capture and 0.5 μg/mL for detection; Stx2e (E167Q) was used at 10 ng/mL in PBS. B) Standard curve for the most sensitive anti-Stx2e sandwich ELISA. Stx2e-3 was used for capture (1 μg/mL); Stx2e-2 was used for detection (0.5 μg/mL). Purified Stx2e (E167Q) toxoid was used as the antigen. The standard curves were linear from 8 to 0.01 ng/mL.

In order to determine which combination of Stx2e mAbs provides the greatest sensitivity for Stx2e, we analyzed all possible combinations of the four Stx2e mAbs that we generated. Almost all combinations were effective at detecting Stx2e (Fig 2A). Although the Stx2e-1/2 and Stx2e-2/1 antibody combinations gave the strongest ELISA signal at a Stx2e concentration of 10 ng/mL (p-value < 0.01), the ELISA comprised of the Stx2e-3/Stx2e-2 combination was the most sensitive due to its lower background, with a limit of detection (LOD) of just 11.8 pg/mL (Fig 2B).

**Fig 3:**
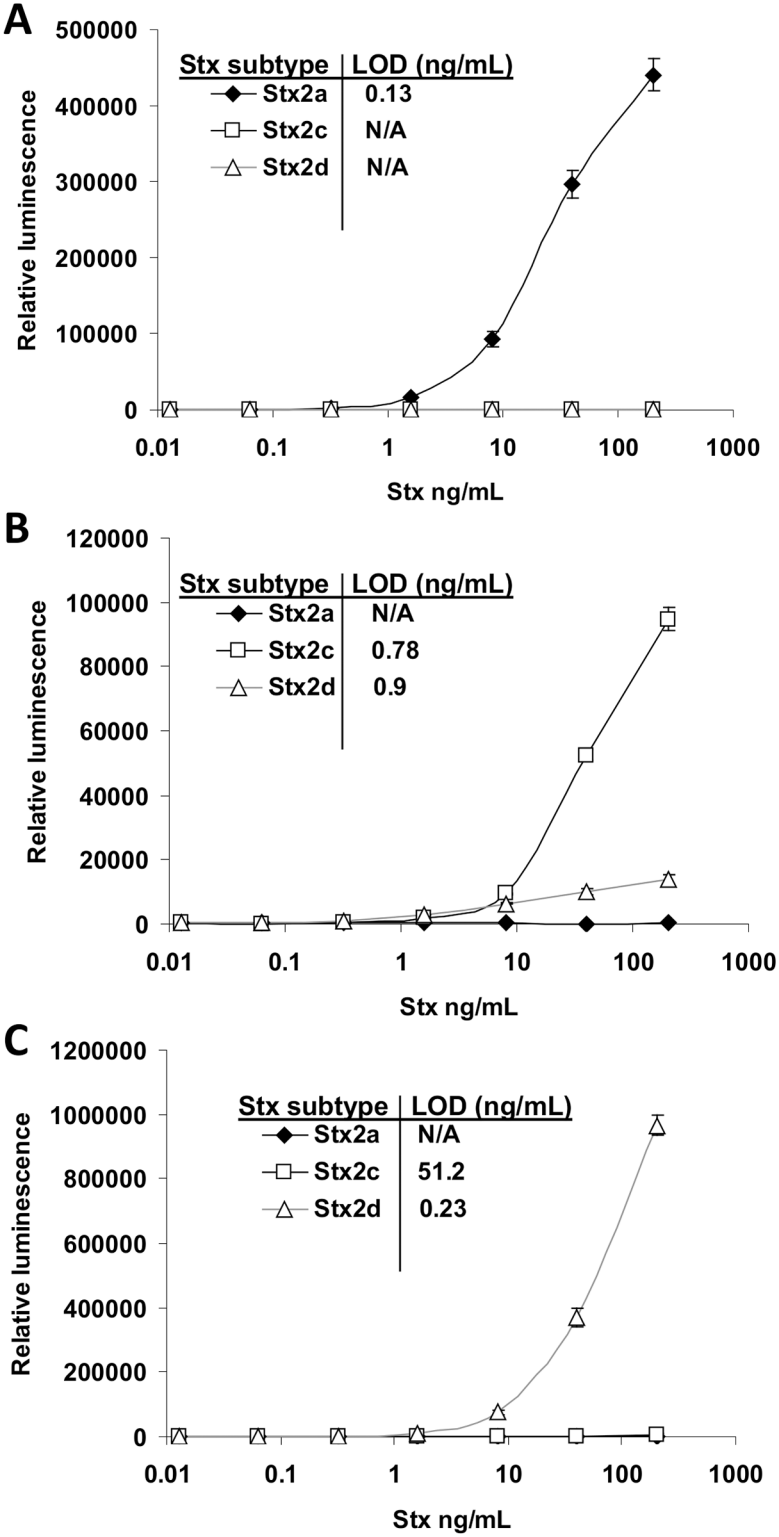
Stx2a-, Stx2c-, and Stx2d-specific ELISAs. A) A Stx2a-specific ELISA (Stx2-5 used for capture; Stx2-1 for detection) was conducted on pure Stx2a, Stx2c, and Stx2d. B) A Stx2c-specific ELISA (Stx2-5 for capture; Stx2e-2 for detection) was conducted on pure Stx2a, Stx2c, and Stx2d. C) A Stx2d-specific ELISA (Stx2e-3 for capture; Stx2e-2 for detection) was conducted on pure Stx2a, Stx2c, and Stx2d.

In an effort to find antibody combinations that could distinguish Stx2 subtypes, four antibodies described in the study were used with two previously described mAbs, Stx2-1 and Stx2-5, derived from Stx2a [13] in ELISAs. All possible antibody combinations using Stx2e-1, Stx2e-2, Stx2e-3, Stx2e-4, Stx2-1, and Stx2-5 for capture and/or detection were evaluated using cell-free media from all seven Stx2 subtypes (pure toxins are currently not available for some subtypes of Stx2). Among the combinations of antibodies, mAbs Stx2-5/Stx2-1 (capture/detection) appeared to recognize only Stx2a (S1 Table). The Stx2-5/Stx2e-2 combination had good affinity for Stx2c and recognized Stx2d weakly, but did not detect Stx2a. The combination of Stx2e-3/Stx2e-2 was very effective at detecting Stx2d and Stx2e, detected Stx2c poorly, and did not recognize Stx2a. These three antibody combinations were then evaluated for their efficacy at distinguishing Stx2a, Stx2c, and Stx2d. Using pure Stx2a, Stx2c, and Stx2d, standard curves were conducted with all three ELISA combinations (Fig 3). As expected, the Stx2-5/Stx2-1 antibody combination was specific to Stx2a only (Fig 3A) (with a LOD of 0.13 ng.mL). The Stx2-5/Stx2e-2 combination recognized Stx2c well (LOD = 0.78 ng/mL), Stx2d poorly (LOD = 0.9 ng/mL), and did not detect Stx2a (Fig 3B). The Stx2e-3/Stx2e-2 combination was very effective at detecting Stx2d (LOD = 0.23 ng/mL), very poor at detecting Stx2c (LOD = 51.2 ng/mL), and did not detect Stx2a at all (Fig 3C).

### Receptor binding and neutralization

**Fig 4:**
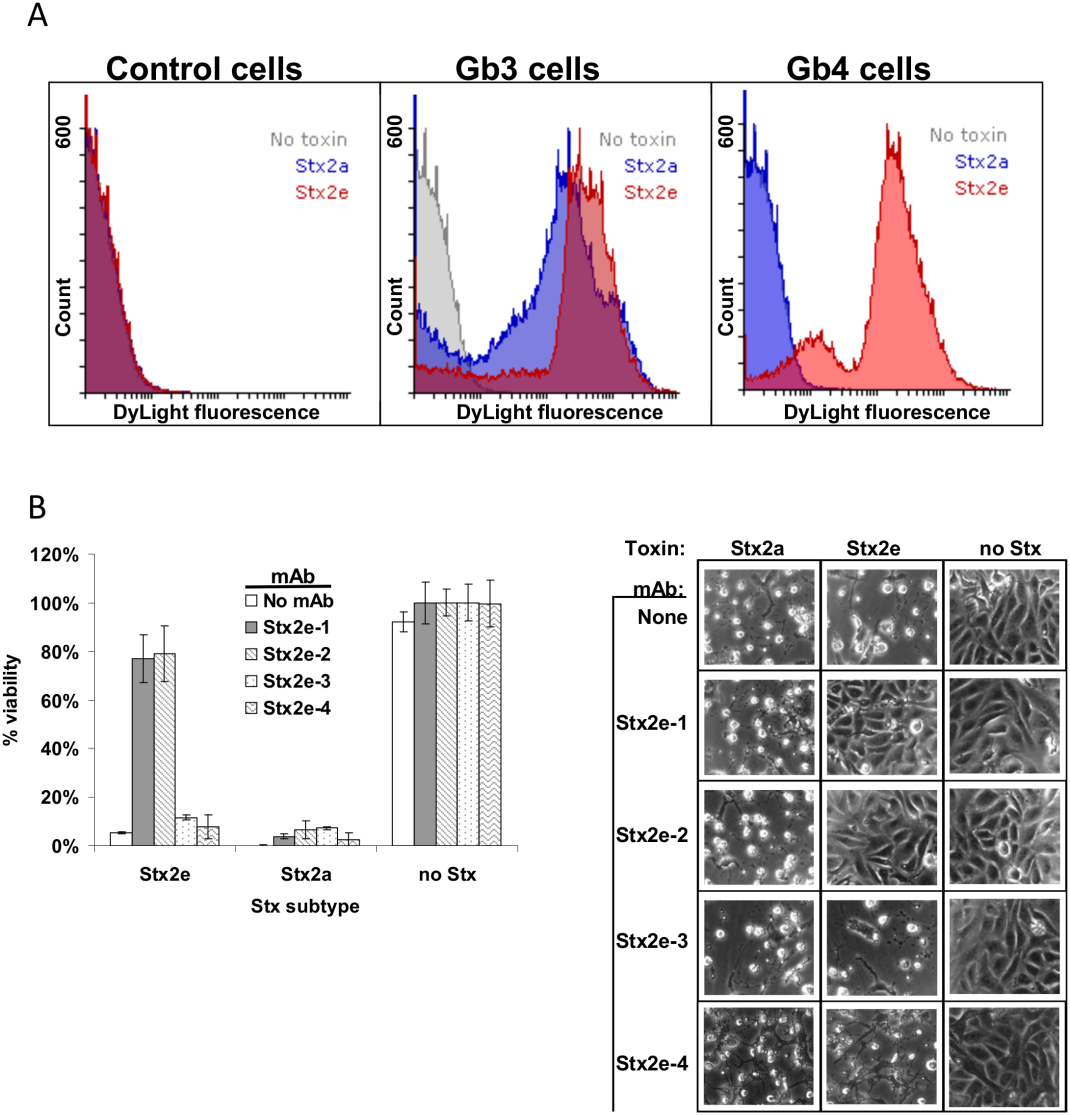
Receptor binding and neutralization of Stx2e. A) FACS analysis of Gb3-LPS, Gb4-LPS, and control *E*. *coli* cells. Stx2e (E167Q) and mAb Stx2e-3 were used at 1 μg/mL, and 50,000 cells were analyzed per sample. B) Administration of mAbs Stx2e-1 or Stx2e-2 at 10 μg/mL protects Vero cells from Stx2e toxicity (Stx2e-containing media at a 10-fold dilution). mAbs Stx2e-3 and Stx2e-4 (also at 10 μg/mL) do not. None of the four mAbs protect Vero cells from Stx2a (Stx2a-containing media at a 10-fold dilution).

The primary cellular receptor for Stx2e is thought to be Gb4-Cer [26]; it has been previously shown, however, that Stx2e can bind a wide range of receptors (Gb3, Gb4, Forsman, etc.) [12]. Using Gb3- and Gb4-LPS-expressing *E*. *coli* and our new mAbs, we sought to confirm receptor specificities for Stx2e. In FACS analysis, Stx2e (E167Q) bound to both Gb3- and Gb4-LPS-expressing cells, but Stx2a (E167Q) only bound to Gb3-LPS-expressing cells (Fig 4A). Antibodies recognizing the B subunits of Stx frequently neutralize the toxin that they are specific to in cell toxicity assays [13, 16]. Our mAbs against Stx2e were not an exception: both B subunit-specific mAbs (Stx2e-1 and Stx2e-2) protected Vero cells from Stx2e toxicity derived from cell-free culture media, whereas both A subunit-specific mAbs (Stx2e-3 and Stx2e-4) did not (Fig 4B).

### Stx2e immunofluorescence

**Fig 5:**
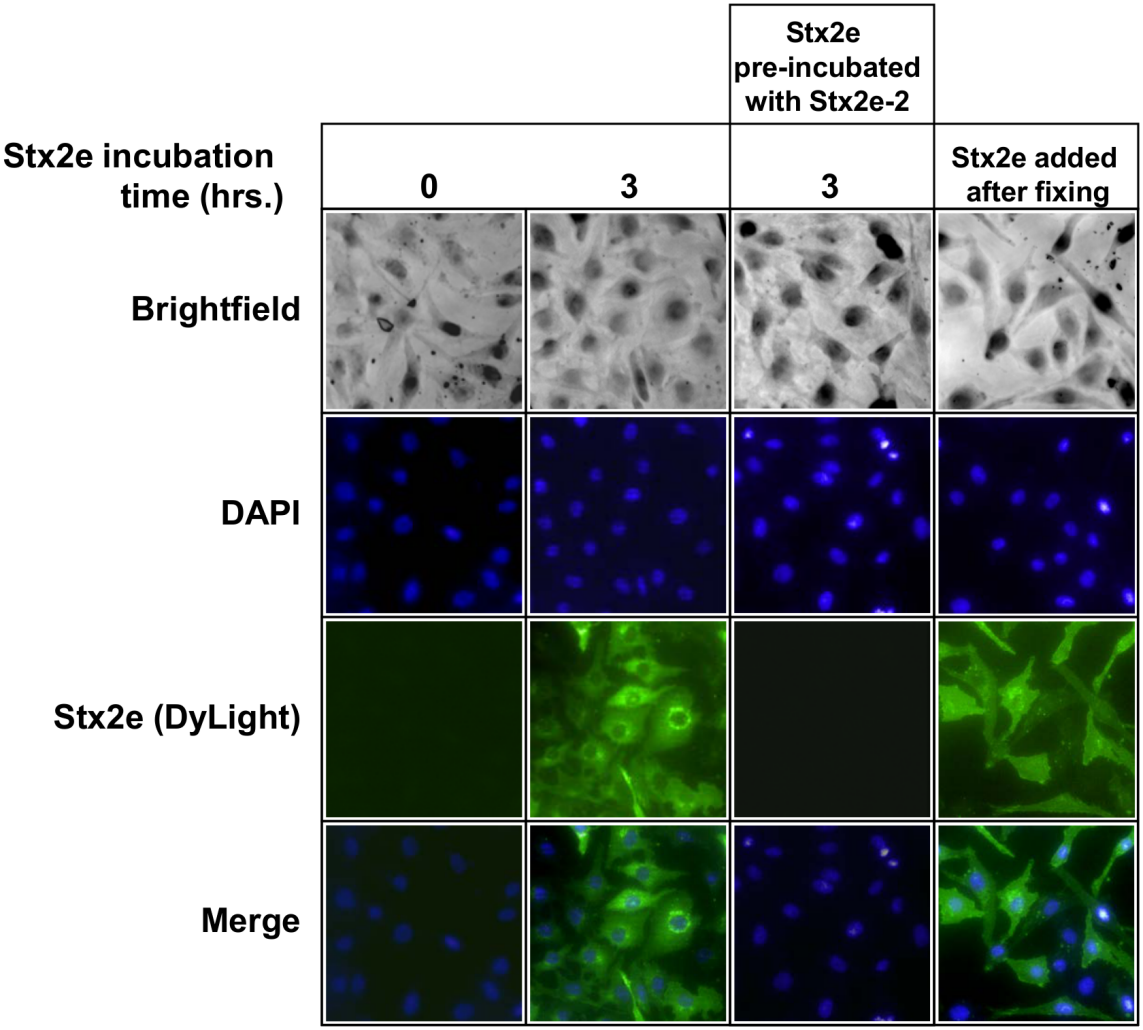
Immunofluorescence of Stx2e (E167Q) in Vero cells. mAb Stx2e-3 (1 μg/mL) was used to visualize Stx2e (E167Q) (1 μg/mL) in Vero cells. Stx2e (E167Q) was pre-incubated with either neutralizing mAb Stx2e-2 in media or media alone before adding to Vero cells. Cells were either fixed in paraformaldehyde before Stx2e (E167Q) treatment or after a 0 or 3 hour incubation with Stx2e (E167Q). After fixing and Stx2e (E167Q) treatment, the cells were blocked with BSA, incubated with mAb Stx2e-3, then incubated with anti-mouse DyLight (green images) and DAPI (to stain nuclei, blue images).

Immunofluoresence is a useful method to track Stx as it progresses from cell surface binding to its target cellular compartments. Interactions of Stx1 and Stx2 with Vero cells have been demonstrated successfully using FITC-labeled anti-mouse IgG secondary antibody [27]. In this study, we demonstrate that the Stx2e (E167Q) toxoid is capable of binding receptors on the surface of Vero cells by first fixing the cells, then treating the cells with the toxoid using immunofluorescence assays. Cells without toxoid treatment were used as a negative control and no green fluorescent signals were observed for these cells (Fig 5, “Stx2e added after fixing” column). To determine if Stx2e (E167Q) toxoid is capable of entering the cells, Vero cells were treated with Stx2e (E167Q) for three hours prior to fixing. As shown in Fig 5, Stx2e (E167Q) entered the cells successfully, and appears to spread throughout the cytoplasm but concentrate around the nucleus. It has been suggested that anti-Stx2e single-chain antibodies can neutralize Stx2e toxicity by blocking the binding of Stx2e to cellular receptors [16]. In agreement with this report, we found that pre-incubation of Stx2e (E167Q) with our neutralizing mAb Stx2e-2 disabled the toxoid binding to Vero cells as well (Fig 5, “Stx2e pre-incubated with Stx2e-2” column). Our immunofluorescence assays also indicate that the mAb Stx2e-3 is able to recognize the Stx2e (E167Q) interacting with cells, regardless of whether the toxoid is internal or external.

## Discussion

In this study, we report the characterization of four new monoclonal antibodies against Stx2e. Two of these (mAbs Stx2e-1 and Stx2e-2) predominantly recognize the B subunits of Stx2e and Stx2f. The others (mAbs Stx2e-3 and Stx2e-4) recognize the A subunits of all Stx2 subtypes except Stx2f. The most sensitive sandwich ELISA incorporating these antibodies included mAb Stx2e-3 for capture and Stx2e-2 for detection, which had a LOD of 11.8 pg/mL for Stx2e. The two B subunit-specific mAbs had a strong ability to neutralize Stx2e toxicities derived from bacterial culture media, while the A subunit-specific mAbs did not. Using these new mAbs and previously developed mAbs against Stx2a, we were able to generate ELISAs that can differentiate between the very closely related and clinically relevant subtypes of Stx2a, Stx2c, and Stx2d.

Although diverse methods have been developed for detecting Stx1 and Stx2 directly, immuno-based assays appear to be the most convenient and reliable. However, Stx2e produced by STEC is undetectable or poorly detected by many major commercial ELISAs and lateral flow assays (Premier, ProspecT, and Ridascreen) [11]. This is likely due to a lack of recognition by the monoclonal antibodies incorporated into these assays [17]. The most sensitive ELISA using the high affinity mAbs developed in this study has a very low limit of detection and detected Stx2e in bacterial culture media. This suggests that this assay may be used to compliment PCR for detecting the presence of viable Stx2e-producing *E*. *coli* in pig feces, food, or water samples, using toxin production rather than the *stx2e* gene as a marker. Sensitivity is extremely critical for Stx2e detection, as Stx2e expression levels vary dramatically from isolate to isolate [2].

Stx2 is an important virulence factor linked to severe human illnesses and molecular typing studies have demonstrated that there is a strong correlation between STEC strains harboring certain Stx2 subtypes and disease severity [28, 29]. In addition, many strains of STEC express both Stx2a and Stx2c or Stx2d: it would be valuable to know which subtype and how much of each subtype is produced by bacterial strains in an unknown sample, and whether Stx2 subtypes may have a synergistic effect upon toxicity. Up until now, all ELISAs for Stx2a cross-react with the very similar Stx2c and Stx2d subtypes, meaning that the immunological discrimination between Stx2a, Stx2c, and Stx2d has been impossible. Our Stx2-5/Stx2-1 ELISA allows for distinction and quantitation of Stx2a apart from Stx2c or Stx2d. Similarly, our Stx2-5/2e-2 ELISA can distinguish Stx2c apart from Stx2a, and our Stx2e-3/2e-2 ELISA can detect Stx2d, but not Stx2a or Stx2c. However, our previous study indicates that hybridization does occur between Stx types present in the same bacterial cells [21], which complicates the interpretation of results obtained from cells expressing two different subtypes of Stx2. For example, if a Stx2a-specific ELISA were performed on a Stx2a/2c expressing STEC strain, the signal would consist of intact Stx2a and Stx2a/2c hybrids. Thus, the amount of pure Stx2a or Stx2c (or that of hybrid toxins) would be unknown. These subtype-specific ELISAs could be useful for analyzing a heterogeneous mixture containing several strains of STEC, each expressing only one Stx2 subtype, however. Eventually, a combination of mass spectrometry and these subtype-specific mAbs, such as Stx2-1 or Stx2e-2, could be used to detect and quantify Stx2a/2c hybrid toxins.

Immunofluorescence could be a valuable tool to study the kinetics of how Stx2e progresses from membrane to cytoplasm in a target cell. In this study, it has already helped confirm the mechanism of neutralization by Stx2e B-subunit antibodies. Moreover, it may provide hints as to when the AB_5_ complex of Stx2e falls apart within a target cell, and where it occurs. Our mAbs Stx2e-2 and Stx2e-3 could be indispensable to these studies. If we could label our A and B subunit-specific antibodies with different fluorescent dyes, we could theoretically monitor the moment when the Stx2e complex falls apart within Vero cells. Analyzing primary or immortalized pig endothelial cells could also shed light on the Stx2e-specific disease process in porcine edema disease.

These new mAbs against Stx2e could satisfy the unmet needs of a sensitive immunoassay for Stx2e and have broad potential applications, since they can recognize both subunits of Stx2e, can distinguish Stx2a from Stx2c and Stx2d when used with our other antibodies in an ELISA, are compatible with immunofluorescence, and possess the ability to neutralize the cytotoxicity of Sx2e.

## Supporting Information

S1 Fig Single-antibody sandwich ELISAs for B-subunit subtype specificity. mAbs Stx2e-1 and Stx2e-2 were used in single-antibody sandwich ELISAs (same antibody for capture and detection) to detect Stx2 subtypes in cell-free media. Media was used at a 10-fold dilution in PBS. Detection antibodies were biotinylated and used at 0.5 ug/mL. Log scale is used due to variation in the amount of each Stx2 subtype present in the media.
(TIF)

S1 File Raw data for quantitative experiments (ELISAs, cell viability assays) is provided here. (XLS)

S1 Table mAb combinations for Stx2 subtype-specific ELISAs. mAb combinations used to distinguish Stx2a, Stx2c, and Stx2d were analyzed for their ability to detect the seven Stx2 subtypes. Cell-free media (at a 10-fold dilution in PBS) from the seven Stx2 subtypes was analyzed.
(DOC)
