# Supplementary figures and images for: New Stx2e Monoclonal Antibodies for Immunological Detection and Distinction of Stx2 Subtypes
Source: PLoS One. 2015 Jul 20;10(7):e0132419. doi: 10.1371/journal.pone.0132419 (PMC4507848; doi:10.1371/journal.pone.0132419)

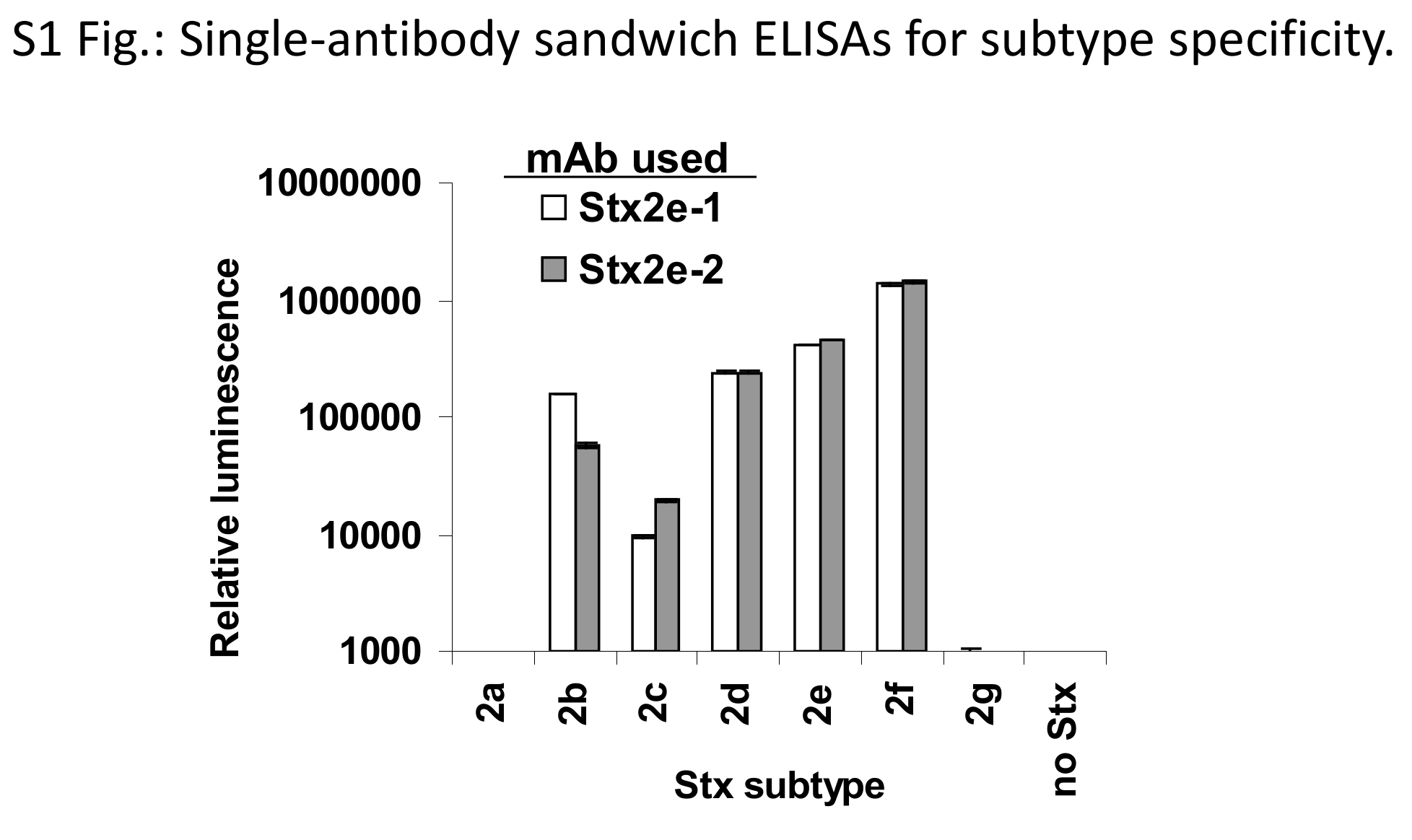

Supplement: S1 Fig — mAbs Stx2e-1 and Stx2e-2 were used in single-antibody sandwich ELISAs (same antibody for capture and detection) to detect Stx2 subtypes in cell-free media. Media was used at a 10-fold dilution in PBS. Detection antibodies were biotinylated and used at 0.5 ug/mL. Log scale is used due to variation in the amount of each Stx2 subtype present in the media. (TIF) [file pone.0132419.s001.tif]
