# Supplementary material for: New Stx2e Monoclonal Antibodies for Immunological Detection and Distinction of Stx2 Subtypes
Source: PLoS One. 2015 Jul 20;10(7):e0132419. doi: 10.1371/journal.pone.0132419 (PMC4507848; doi:10.1371/journal.pone.0132419)
Supplement: S1 Table — mAb combinations used to distinguish Stx2a, Stx2c, and Stx2d were analyzed for their ability to detect the seven Stx2 subtypes. Cell-free media (at a 10-fold dilution in PBS) from the seven Stx2 subtypes was analyzed. (DOC) [file pone.0132419.s003.doc]

**S1 Table: Stx2 specificies by ELISA pair.**

| **mAb** | | **Stx2 subtype** | | | | | | | |
| --- | --- | --- | --- | --- | --- | --- | --- | --- | --- |
| **Capture** | **Detection** | **a** | **b** | **c** | **d** | **e** | **f** | **g** | **No Stx** |
| Stx2-5 | Stx2-1 | +++ | - | - | - | - | - | - | - |
| Stx2-5 | Stx2e-2 | - | - | ++ | +? | - | - | - | - |
| Stx2e-3 | Stx2e-2 | - | + | +? | ++++ | +++ | - | - | - |

| Symbol | Relative luminescence |
| --- | --- |
| +? | 10,000 - 50,000 |
| + | 50,000 – 200,000 |
| ++ | 200,000 – 500,000 |
| +++ | 500,000 – 1,000,000 |
| ++++ | 1,000,000+ |
